# Plant species richness and composition of a habitat island within Lake Kastoria and comparison with those of a true island within the protected Pamvotis lake (NW Greece)

**DOI:** 10.3897/BDJ.8.e48704

**Published:** 2020-01-15

**Authors:** Alexandros Papanikolaou, Maria Panitsa

**Affiliations:** 1 Division of Plant Biology, Department of Biology, University of Patras, Patras, Greece Division of Plant Biology, Department of Biology, University of Patras Patras Greece

**Keywords:** protected area, Natura 2000, plant diversity, α-diversity, β-diversity, spatial turnover, monitoring

## Abstract

Lake Kastoria is one of the potentially “ancient” Balkan lakes that has a great environmental importance and ecological value, attracts high touristic interest and is under various anthropogenic pressures. It belongs to a Natura 2000 Special Protection Area and a Site of Community Interest. The city of Kastoria is located at the western part of the lake and just next to it, towards the centre of the lake, is a peninsula, a habitat island. In the framework of research concerning the flora of lake islands of Greece, one of the main objectives of the present study is to fill a gap concerning plant species richness of the habitat island within the protected Lake Kastoria, which is surrounded by the lake except for its north-western part where the border of the city of Kastoria is located. Floristic analysis of the habitat island of Lake Kastoria is in large measure accounted, concerning chorology with emphasis on Balkan endemics (8.7%), life forms, by hemicryptophytes (36.1%), therophytes (33.2%), phanerophytes (16.4%) and geophytes (10.9%) and, for habitats, by taxa preferring agricultural and ruderal ones (53.3%). Another objective is to compare its floristic composition to the one of the island within the protected urban Lake Pamvotis - one of the very few lake islands in Greece - focusing on the influence of urbanisation. The α- and β- diversity are measured in order to reveal floristic differences. Beta diversity partitioning in turnover and nestedness showed that the β-diversity is mostly expressed as compositional turnover. The role of the society in combination with long-term programmes for the study of plant species richness, functional diversity and patterns of species assemblages over time are necessary for the effective management and protection of protected areas, including lake insular areas.

## Introduction

The majority of ancient or putatively ancient European lakes is restricted to the Balkan area and is thought to be restricted within a cluster of lakes, about 300 km around Lakes Ohrid and Prespa (Touka et al. 2018), including lakes such as the lakes Skutari (Montenegro, Albania), Mikri Prespa (Greece, Albania), Vegoritis, Kastoria or Orestias, Pamvotis and Trichonis (Greece). Lake Kastoria or Orestias is one of the potentially “ancient” lakes of the Balkans, meaning long-lived modern or palaeo-lakes (Frogley et al. 2001, Frogley et al. 2002, Frogley and Preece 2004). Lake Pamvotis is an ancient lake of Pleistocenic biota, characterised as a Quaternary refugium (Frogley et al. 2001, Tzedakis 2002).

**Figure 1.**
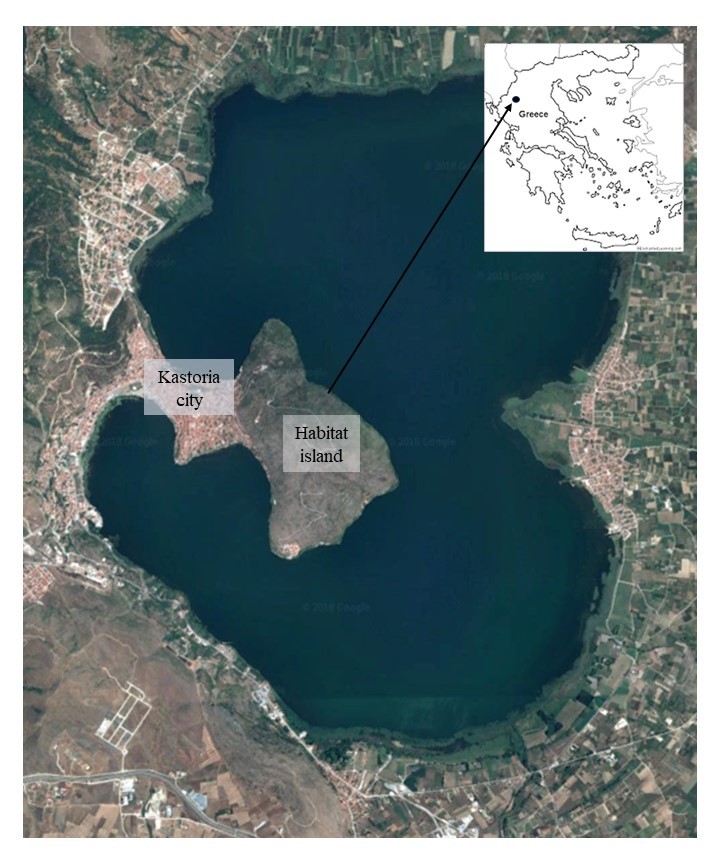
Map of the habitat island within Lake Kastoria.

Lake Kastoria, belongs to the Natura 2000 Special Protection Area GR1320003 and Site of Community Importance GR1320001 (Fig. 1) and it falls under the responsibility of the Protected Areas of Western Macedonia Management Body. Lake Pamvotis belongs to the Natura 2000 Special Protection Area and Site of Community Importance GR2130005 and it falls under the responsibility of the Lake Pamvotis Management Body (see Sarika et al. 2019). Kastoria and Pamvotis lakes, both urban lakes, are rather heavily modified (Latinopoulos et al. 2018). Considering the fact that Lake Kastoria is an urban lake with a population of 17,000 inhabitants living on the shoreline and 35,000 in the entire catchment, it is easy to understand that this lake is affected by human activity. This is also the case for Lake Pamvotis, near which Ioannina city is located with a population of more than 120,000 inhabitants (Kati et al. 2006, Sarika et al. 2019).

Both lakes have attracted research interests as sedimentary archives on long term environmental and climate history and as a hotspot for European biodiversity (Touka et al. 2018). Many studies have also been published, concerning the hydrology and hydromorphology, the assessment of the typology and the trophic status, the plankton, the hydrophyte vegetation of North-western Greece's lakes, focusing on the lakes of Kastoria and Pamvotis (amongst others Koussouris et al. 1987, Latinopoulos et al. 2016, Latinopoulos et al. 2018, Moustaka-Gouni et al. 2006, Papastergiadou et al. 2009, Stefanidis and Papastergiadou 2007, Stefanidis and Papastergiadou 2010, Katsiapi et al. 2016, Kagalou and Psilovikos 2014, Moustaka-Gouni et al. 2007).

Lakes are “negative islands”, that is, they are more or less isolated freshwater areas surrounded by a hostile land matrix, behaving as islands in many biogeographical and ecological respects (Whittaker and Fernandez-Palacios 2007). Since lakes behave like that, any type of island (true or habitat) within them is a lake island in a negative island. Habitat islands are essentially all forms of insular systems that do not qualify as being “real islands”. They are discrete patches of distinct terrestrial habitat surrounded by strongly contrasting habitats (Whittaker and Fernandez-Palacios 2007). Within both studied lakes, there are islands. A true island can be found within Lake Pamvotis (Sarika et al. 2019) and a habitat island, a peninsula, within Lake Kastoria. This habitat island, neighbouring the city of Kastoria, is still floristically unexplored and is surrounded by the lake apart from its north-western part where the border of the city is located (Fig. 1). Human interference is obvious both in the protected lakes as well as in the two lake islands. Thus, it should be noted that the need for monitoring species diversity in protected areas is urgent and the assessment of species diversity is crucial (Chelli et al. 2019). In order to understand the patterns of species richness, they can be separated into different components: α-diversity that represents the species richness of a local habitat and β-diversity that is the component that represents the difference in species composition between sample units or between habitats and can be driven by nestedness that reflects a non-random process of disaggregation of assemblages and turnover that is a process of substitution of species by environmental selection or historical and spatial restriction (Whittaker 1965, Coelho et al. 2018, Baselga 2010).

In the framework of this study concerning plant species richness of lake islands of North-Western Greece, the main objectives are a) to fill the gap in the floristic information available concerning the study of the floristic composition of the habitat island within the protected Lake Kastoria and b) to depict the differences in species richness and composition with those of the island within the protected Pamvotis lake (Sarika et al. 2019), measuring α-diversity and estimating β-diversity through the contribution of spatial turnover and nestedness and focusing on the influence of urbanisation, since both are within urban lakes.

## Material and methods

### Survey area

Lake Kastoria has a surface area of 27.9 km^2^ at an altitude of 630 m in the Kastoria Prefecture, North-Western Greece. It has an average depth of 4.4 m and a maximum depth of 9.1 m. The city of Kastoria (> 47,160 inhabitants) is located on the western part of the lake and discharges from there have resulted in increased eutrophication (Moustaka-Gouni et al. 2007). Lake Kastoria is a very fragile shallow aquatic ecosystem, long challenged by the various rural (logging, agricultural wastes, stockbreeding etc.), craft (tanneries, fur/leather production) and urban (e.g. sewer discharges, rubble depositions and extensive littering) activities of the area. The area is a significant touristic destination, especially the caves that have been formed (Dragon Cave), (Kagalou and Psilovikos 2014). The eastern border of the city of Kastoria forms the north-western border of the habitat island studied (**KaHI**), the peninsula, that is surrounded by the lake at all its other parts (Fig. 1). Human intervention is evident with urban development dominating in Lake Kastoria (Kagalou and Psilovikos 2014, Kati et al. 2006).

Lake Pamvotis has a surface area of 22 km^2^ at an average altitude of 470 m. It is a shallow lake (mean depth of 4.3 m) and the city of Ioannina (>120,000 inhabitants) is located at its south-western part. Although ecologically important, the ecosystem of the lake is seriously aﬀected by organic (Kagalou et al. 2003) and heavy metal (Papagiannis et al. 2004) pollution, riparian habitat urbanisation and hydrological regime disruption. For the purpose of this research, the temporal turnover of plant species richness of the island within Lake Pamvotis (**PaI**) that attracts >170,000 visitors every year, has been studied (Sarika et al. 2019).

### Data Collection and Floristic analysis

The present study concerns plant species richness and composition of the habitat island within Lake Kastoria (KaHI) and comparison with those of the true island within the protected Pamvotis lake (PaI). It is based on our team's (a) fieldwork at KaHI, from April 2018 to October 2019 and (b) published research concerning plant species diversity of PaI, the island within Lake Pamvotis (Sarika et al. 2019, see sampling period Β), from May 2018 to April 2019. Sample collections on KaHI and PaI during 2018-2019 were carried out by Α. Papanikolaou and Μ. Panitsa and plant specimens are deposited in UPA Herbarium. The sampling scheme used included fieldwork (plant specimen collections and observations) all over KaHI during all seasons of the year and this was also the case for PaI. For the determination of the plant material, Tutin et al. (1964), Tutin et al. (1972), Tutin et al. (1976), Tutin et al. (1980), Tutin et al. (1981), Strid and Tan (1997) and Strid and Tan (2002) were used. The families, genera, species and subspecies are listed, within the major taxonomic groups in alphabetical order. Nomenclature, life form, chorology, status categories and habitat preferences of plant taxa follow Dimopoulos et al. (2013). The six main life forms have been used for the floristic analysis as were given by Dimopoulos et al. (2013) and are summarised as follows: phanerophytes (P), chamaephytes (Ch), hemicryptophytes (H), geophytes (G), therophytes (T) and aquatics (Aq). Chorological categories follow the system of Dimopoulos et al. (2013) and for the analysis, they have been grouped to 5 main chorological elements: Widespread, Mediterranean, Balkan, Greek endemics and Alien.

In the framework of the authors' research, a list of all the information recorded has been created, including the plant taxa registered on KaHI and those of PaI (Sarika et al. 2019), their biological and chorological types and their habitat preferences according to the data provided by the “Vascular Plants of Greece" (Dimopoulos et al. 2013; see also "Flora of Greece web" 2017+, http://portal.cybertaxonomy.org/flora-greece-intro).

The α-diversity is one of the components of species richness, measured as the number of species occurring in a sample unit. In this study, the sample unit is the habitat island (surface area of 27.9 km^2^) and its α-diversity is compared with that of the island within Pamvotis lake (surface area of 22 km^2^). To allow floristic comparisons between KaHI and PaI, a table presenting floristic similarities was prepared. The chi-square test, which is a well-suited statistical tool for these purposes, was used to compare biological and chorological spectra between KaHI and PaI. In order to test β-diversity between them, we checked its two components: spatial species turnover using Sørensen dissimilarity index (b_sor_) and nestedness of assemblages using nestedness-resultant dissimilarity (b_nes_) index (Baselga 2010). The Sørensen dissimilarity index (bsor) is one of the most used measures due to its dependence on the proportion of species shared between two communities, that incorporates both true spatial turnover and differences in richness (Koleff et al. 2003). The Sørensen dissimilarity index (bsor) is formulated as: \begin{varwidth}{50in}
        \begin{equation*}
            bsor = {b+c \over 2a+b+c}
        \end{equation*}
    \end{varwidth} where a is the number of species common to both sites, b is the number of species that occur in the ﬁrst site, but not in the second and c is the number of species that occur in the second site, but not in the ﬁrst. The nestedness-resultant dissimilarity (bnes) index is a measure of the dissimilarity of communities due to the effect of nestedness patterns (Baselga 2010) and is formulated as: \begin{varwidth}{50in}
        \begin{equation*}
            bnes = {max(b,c)-min(b,c) \over 2a+min(b,c)+max(b,c)}*{a \over a+min(b,c)}
        \end{equation*}
    \end{varwidth}

## Results

There is no previous documented information concerning plant species richness of the habitat island within the protected lake of Kastoria studied. Field investigations on the island revealed a total number of 274 plant taxa recorded, out of which 2 are Pteridophyta, 4 Gymnospermae and 268 Angiospermae, belonging to 196 genera and 74 families (Table 1).

**Figure 2.**
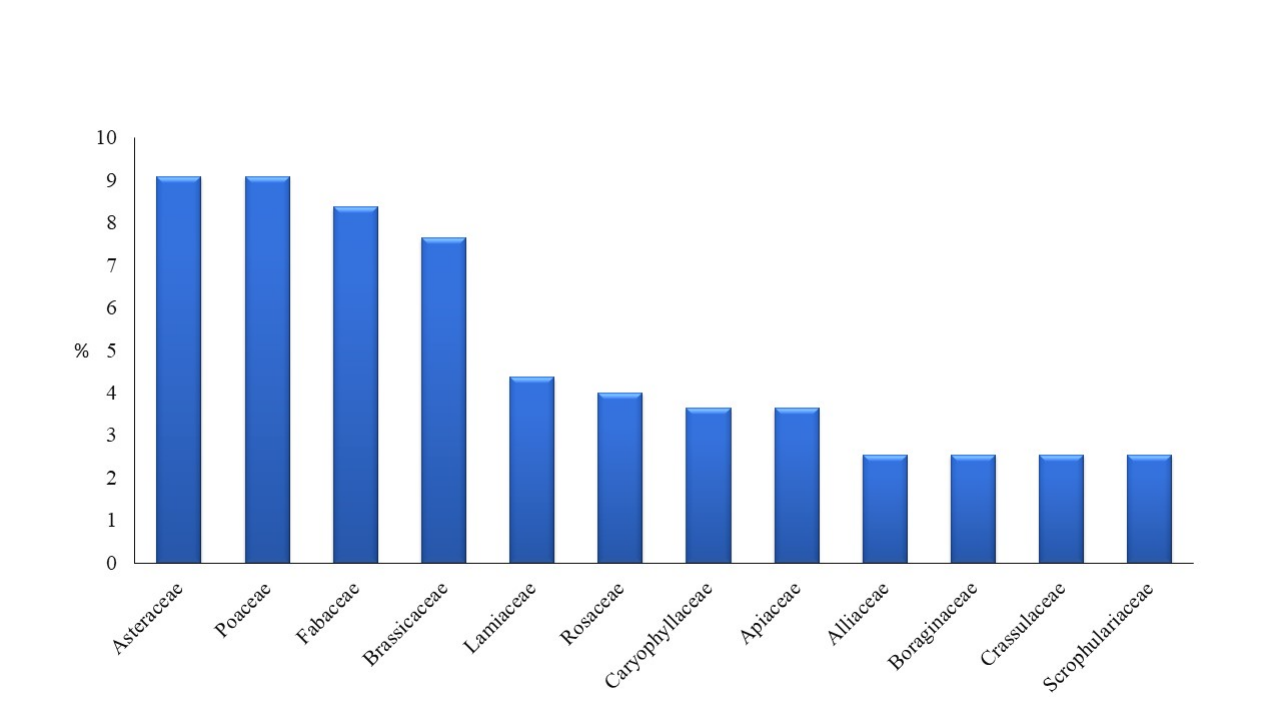
Richest in taxa families of the habitat island within Lake Kastoria.

**Figure 3.**
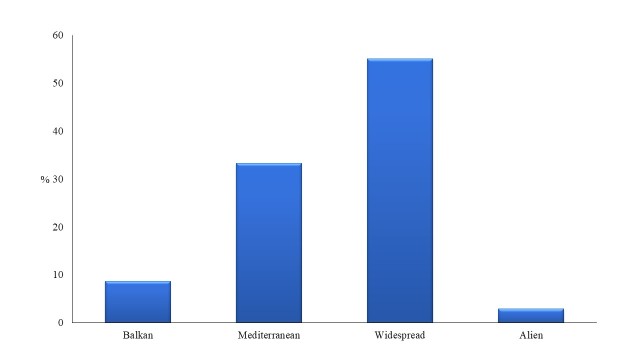
Richest in taxa genera of the habitat island within Lake Kastoria.

Asteraceae (25 taxa), Poaceae (25 taxa), Fabaceae (23 taxa), Brassicaceae (21 taxa), Lamiaceae (12 taxa), Rosaceae (11), Caryophyllaceae (10) and Apiaceae (10 taxa) are the richest in taxa families in the total of plant taxa recorded. Taxa belonging to these families represent 50% of the total flora of the habitat island (Fig. 2). The richest in taxa genera are: *Allium* (6 taxa), *Prunus* and *Medicago* (5 taxa), *Euphorbia, Galium*, *Geranium*, *Silene, Trifolium* and *Poa* (4 taxa each), (Fig. 3).

**Figure 4.**
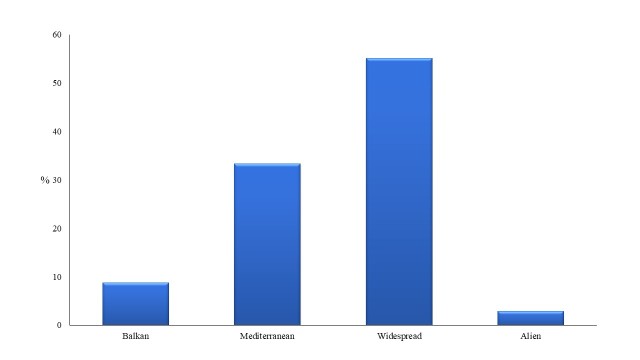
Proportion of chorological categories of the plant taxa diversity of the habitat island within Lake Kastoria.

Regarding the chorological origin of the taxa, widespread taxa dominate (55.1%), followed by Mediterranean elements (33.3%), Balkan taxa (8.7%) and alien taxa, xenophytes (2.9%). Fig. 4 shows proportions of different chorological elements recorded. The presence of Balkan elements is significant, since 24 taxa have been registered (Table 1). Eight naturalised xenophytes have been registered, belonging to 7 families, namely *Ailanthus altissima, Amaranthus quitensis, Broussonetia papyrifera, Cuscuta campestris, Erigeron canadensis, Morus alba, Prunus dulcis* and *Robinia pseudoacacia*.

According to the IUCN Red List (IUCN 2019), *Allium bornmuelleri* is a range-restricted Balkan endemic, characterised as Data Deficient as also are *Allium cyrilli* and *Prunus webbii*, while *Allium amethystinum, A. sphaerocephalon, Prunus avium, P. spinosa* and *Dictamnus albus* are characterised as of Least Concern. *Lilium candidum*, has been assessed as Near Threatened in the national red list (Phitos et al. 2009).

**Figure 5.**
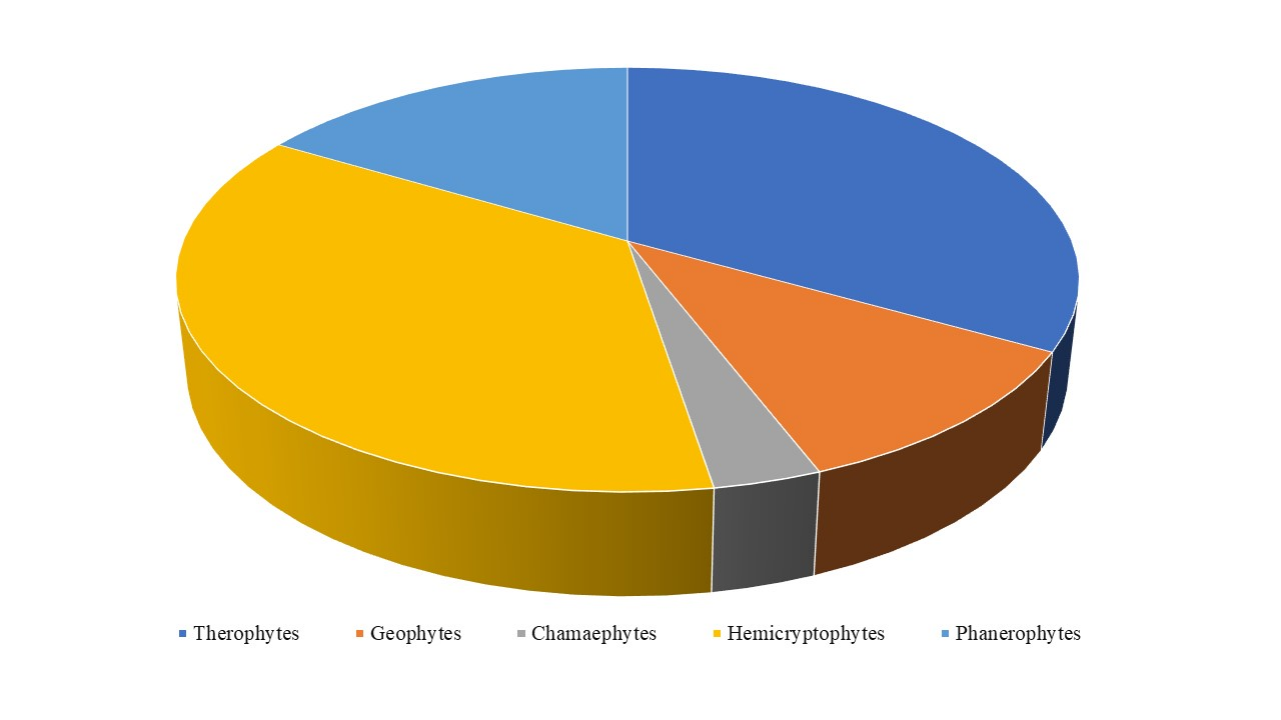
Life form spectrum of the plant diversity of the habitat island within Lake Kastoria.

**Figure 6.**
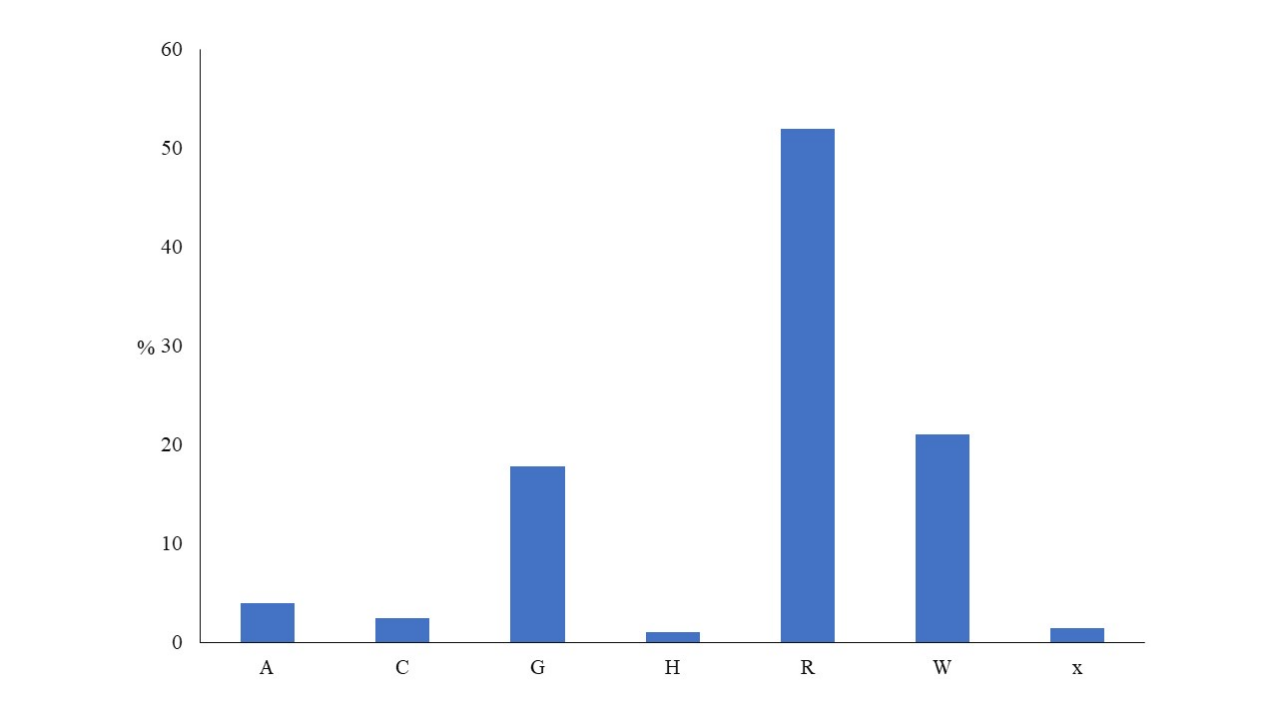
Proportion of different habitat categories for taxa recorded in the habitat island within lake Kastoria: Freshwater habitats (A), Cliffs and rocks (C), temperate and submediterranean grasslands (G), High mountain vegetation (H), agricultural and ruderal habitats (R) and Woodlands and scrub (W). x = generalist taxa.

On the life form spectrum, Hemicryptophytes are the most dominant (36.1%), followed by Therophytes (33.2%), Phanerophytes (16.4%) and Geophytes (10.9%) (Fig. 5). The evaluation of habitat preferences of plant taxa reveals that plants (exclusive or non-exclusive) of agricultural and ruderal habitats (52%) are the most common, followed by plants of grasslands (17.8%), of woodland and scrub (21.1%), exclusively of cliffs and rocks (2.5%) and of freshwater habitats (4%) (Fig. 6).

**Figure 7.**
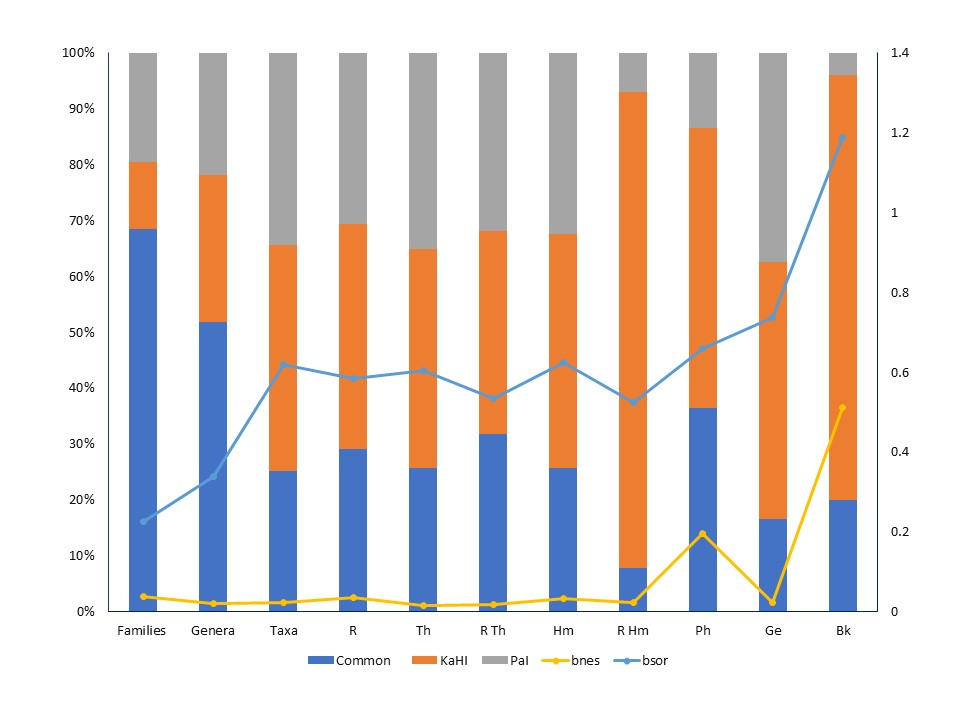
Contribution of different species richness variables of the habitat island within Lake Kastoria (KaHI) and of the island of Lake Pamvotis (PaI), on the beta diversity components: spatial turnover (bsor) and nestedness (bnes). Abbreviations: R = ruderal taxa, Th = Therophytes, Hm = Hemicryptophytes, Ph = Phanerophytes, Ge = Geophytes, Bk = Balkan taxa.

The α-diversity for KaHI is 9.9 per km^2^ and for PaI 10.5 per km^2^. Comparing their floristic composition, 105 taxa were common (25.2%), 169 (40.5%) were registered only on KaHI and 143 (34.3%) only on PaI (Sarika et al. 2019) during their second sampling period from May 2018 to April 2019 (Fig. 7). Using the chi-square test, the observed data of biological and chorological spectra of KaHI and PaI were compared to a null hypothesis that distributes the data according to the expectation that they are due to chance. The observed data do not fit the model, since for biological types: df = 4, x^2^ = 0.457 and p = 0.977 and for chorological types: df = 3, x^2^ = 0.002 and p = 0.999, thus proving the null hypothesis correct and the relation of the data not statistically significant.

Concerning beta diversity, the values of its components, b_sor_ and b_nes_ are presented in Fig. 7. Values of b_nes_ are lower than the values of b_sor_ for all species richness variables. The values of b_sor_ are higher than 0.5 on different functional categories as life forms (therophytes, hemicryptophytes, geophytes), chorological elements (Balkan taxa) and habitat preferences (ruderal taxa, ruderal therophytes). Of the common taxa, 61.9% were ruderal, 3.8% were alien (*Robinia pseudoacacia, Erigeron canadensis, Ailanthus altissima, Cuscuta campestris*) and 5.7% were Balkan and range restricted (*Centaurea graeca, Alyssum chalcidicum, Silene ungeri, Ballotanigra* subsp. *sericea, Ophrys helenae* and *Scabiosa tenuis*).

## Discussion

Lake Kastoria, one of the potentially “ancient” Balkan lakes, is a protected lake of a great environmental importance and ecological value. Lake Kastoria’s habitat island and Lake Pamvotis’ island (both in NW Greece) are two of the very few lake islands occurring in Greece – together with the small islands of Agios Achilleios and Vidronisi within Prespa Lake (N Greece) - all belonging to protected lake areas.

There has been a gap so far concerning plant species richness of the habitat island studied, which is included in the protected area of Lake Kastoria, (GR1320001 and GR1320003). During field investigations conducted on the island, 274 plant taxa were registered in total, belonging to 74 families and 196 genera and presenting a rather high α-diversity. Of the taxa recorded, in total, 38.7% belong to the families Asteraceae, Poaceae, Lamiaceae, Fabaceae and Brassicaceae, as is also the case with the island within Lake Pamvotis (Sarika et al. 2019).

The combination of the high percentage of Mediterranean taxa and the high percentage of therophytes, reflects the Mediterranean character of the flora of the studied habitat island as well as of the Lake Pamvotis island. The proportion of alien taxa is about 2.9%, less than the one recorded for the Greek flora as a whole (5% according to Arianoutsou et al. 2010; 3.8% according to Dimopoulos et al. 2013), but in a restricted area.

The α-diversity, the diversity at a local scale of the two lake islands, was rather similar. Panitsa and Tzanoudakis (2010) stated that the number of species per surface unit (alpha-diversity) is an important parameter that highlights the role of small islands in the conservation of the diversity. The Echinades islet group (Ionian area, Western Greece), hosts a high number of taxa per surface unit (Iliadou et al. 2014), as is also the case in the East Aegean islet groups of Arki and Lipsi (Panitsa and Tzanoudakis 2001). Concerning island biogeography, species richness on different islands is likely to be particularly affected by species richness on the mainland and the degree of inter-island dispersal (Bunnefeld and Phillimore 2012).

The two lake islands studied present remarkable similarities as well as significant differences in their floristic composition. Both islands are located within urban lakes in NW Greece, have a small surface area, are of strong continental character and they also share the same rather strong human strains. Cody (2006) showed that plant species turnover is a highly variable phenomenon, depending on the functional traits of each species, the local geomorphology and the ecological conditions of the studied areas. Urban development fragments, isolates and degrades natural habitats; it also simplifies and homogenises species composition, causing a more linear decline of richness with increasing urbanisation in regions where anthropogenic impact outside cities is more limited (Luck and Smallbone 2010, Norton et al. 2016, Oliveira Hagen et al. 2017). The restructuring of biotic communities in urban areas is non-random and strongly associated with the loss of species, with limited tolerance to urban development and increased abundances of more tolerant species, with urban environments ﬁltering species according to their ecological and life-history traits (amongst others Fischer et al. 2015, Oliveira Hagen et al. 2017). This ﬁltering process occurs across all three stages of the biotic urbanisation process, i.e. arrival, adaptation and spread (Evans et al. 2010). Urbanisation changes seed dispersal functions which promote invasive plant species over closely-related native species (Caughlin et al. 2016).

Ruderal plant taxa are typically occurring and prevailing in disturbed areas, in agricultural and ruderal habitats and especially in sites with pronounced direct or indirect human activity, rural and urban sites, roadsides, excessively grazed and trampled sites, as well as in naturally nutrient-rich and frequently disturbed pioneer habitats (Dimopoulos et al. 2013). On the contrary, species that do not make much use anthropogenic resources, but are largely reliant on the natural habitat lying within a matrix of urban development, face the challenge of maintaining movement through an increasingly fractured landscape to access remnant patches of suitable habitat and have to adapt to declining habitat quality and encroachment of weeds, as well as to the presence of introduced feral and domestic predators within these habitat patches (Bryant et al. 2017). The high percentage of common ruderal taxa between the two lake islands reveals their contribution to their plant species composition, mainly due to the influence of the nearby urban ecosystems and due to human activities in the studied area, in general. Human activities are amongst the factors enhancing turnover (Panitsa et al. 2008) and ruderal flora and vegetation generally represent one of the most dynamic floristic-vegetation complexes (Pyšek et al. 2017).

Beta diversity partitioning in turnover and nestedness showed that the β-diversity of the studied two, habitat and true, islands was mostly expressed as compositional turnover since the nestedness component was lower than the turnover one. This result was mostly based on changes in turnover rates (such as changes in floristic composition), rather than a change due to nestedness. Changes in community composition and spatial structure (spatial beta diversity) need a lot of work to be quantified (Dornelas et al. 2014, McGill et al. 2015) and focusing on the composition of plant species richness of the two lake islands showed that human interference is continuous and the values of the spatial turnover component of β-diversity are significant. A high percentage of common taxa between the two islands are ruderal therophytes and hemicryptophytes but they still conserve floristic dissimilarity concerning Balkan endemic taxa. That means that functional diversity indices allow the quantiﬁcation and comparison of functional diversity amongst communities much more than simple measurements of biodiversity (Pla et al. 2012).

Both protected lakes and the islands within them suffer because of various anthropogenic pressures affecting their plant species and functional diversity (Latinopoulos et al. 2016, Latinopoulos et al. 2018, Papastergiadou et al. 2009, Stefanidis and Papastergiadou 2007, Stefanidis and Papastergiadou 2010). Since they are urban lakes, urbanisation significantly influences their ecosystems’ functioning and the services they provide to humans (Alberti 2016, Alberti et al. 2003). Consequently, the role of society on effective management and conservation of these urban affected islands will be crucial. Additionally, long-term programmes for the study of plant species richness, functional diversity and patterns of species assemblages over time in protected areas, as in the studied area, is more than necessary (Chelli et al. 2019) for their effective management and protection.

## Figures and Tables

**Table 1.** List of plant taxa recorded on the habitat island within Lake Kastoria. Abbreviations: **Bk** = Balkan chorological element, **X** = alien, xenophyte, non-native plant taxa including cultigens, permanently established in at least one floristic region of Greece, **r** = range-restricted plant taxon characterised by a restricted distribution, by populations occurring along a linear distance not exceeding 500 km and **R** = ruderal taxon

**Plant Families**	**Plant taxa**	**Bk**	**Status**	**R**
**Pteridophytes**				
** Aspleniaceae **	*Asplenium ceterach* L.			
**Dennstaedticeae**	*Pteridium aquilinum* (L.) Kuhn			
**Gymnosperms**				
** Cupressaceae **	*Cupressus sempervirens* L.			
	Juniperus oxycedrus subsp. deltoides (R.P. Adams) N.G.Passal.			
** Ephedraceae **	*Ephedra foeminea* Forskål			
** Pinaceae **	*Pinus brutia* Ten. (cultivated)			
**Angiosperms**				
** Acanthaceae **	*Acanthus hungaricus* (Borbás) Baen.	*		
** Aceraceae **	*Acer platanoides* L.			
	*Acer pseudoplatanus* L.			
** Alliaceae **	*Allium amethystinum* Tausch			1
	*Allium bornmuelleri* Hayek	*	r	
	*Allium cyrilli* Ten.			1
	*Allium guttatum* Steven			
	*Allium rhodopeum* Velen.			1
	*Allium sphaerocephalon* L.			1
** Amaranthaceae **	*Amaranthus quitensis* Kunth		X	1
** Amaryllidaceae **	*Sternbergia lutea* (L.) Sprend			
** Anacardiaceae **	*Pistacia terebinthus* L.			
** Apiaceae **	Anthriscus sylvestris subsp. nemorosus (M.Bieb.) Koso-Pol.			1
	*Daucus carota* L.			1
	*Eryngium campestre* L.			1
	*Foeniculum vulgare* Mill.			1
	*Malabaila involucrata* Boiss. & Spruner	*		1
	*Oenanthe aquatica* (L.) Poir.			
	*Orlaya daucoides* (L.) Greuter			1
	*Pastinaca hirsuta* Pančić	*		1
	Scandix australis subsp. grandiflora (L.) Thell.			1
	*Tordylium apulum* L.			1
** Apocynaceae **	Nerium oleander L. subsp. oleander (obs.)			
** Araceae **	Arum italicum Mill. subsp. italicum			
	*Arum maculatum* L.			
** Araliaceae **	*Hedera helix* L.			
** Aristolochiaceae **	*Aristolochia clematitis* L.			1
** Asclepiadaceae **	*Cionura erecta* (L.) Griseb.			1
** Asparagaceae **	*Asparagus acutifolius* L.			
** Asphodelaceae **	*Asphodeline lutea* (L.) Rchb.			
** Asteraceae **	*Achillea clypeolata* Sm.			
	*Achillea millefolium* L.			
	*Anthemis arvensis* L.			1
	*Artemisia vulgaris* L.			1
	*Calendula arvensis* (Vaill.) L.			1
	Carduus nutans subsp. leiophyllus (Petrović) Stoj. & Stef.			1
	*Carduus pycnocephalus* L.			1
	*Centaurea graeca* Griseb.	*	r	
	*Cichorium intybus* L.			1
	*Crepis neglecta* L.			
	*Echinops sphaerocephalus* L.			1
	*Erigeron canadensis* L.		X	1
	*Lactuca muralis* (L.) Gaertn.			
	Lactuca viminea (L.) J. Presl & C. Presl subsp. viminea			
	Leontodon hispidus L. subsp. hispidus			
	*Matricaria recutita* L.			1
	*Podospermum canum* C.A.Mey.			
	*Senecio vernalis* Waldst. & Kit.			1
	*Senecio vulgaris* L.			1
	*Silybum marianum* (L.) Gaertn.			1
	*Sonchus oleraceus* L.			1
	Taraxacum sect. Fontana Soest			
	Taraxacum sect. Ruderalia Kirschner & al.			1
	Tragopogon porrifolius subsp. eriospermus (Ten.) Greuter			1
	*Xanthium strumarium* L.			1
** Boraginaceae **	Anchusa officinalis subsp. intacta (Griseb.) Selvi & Bigazzi			1
	*Cynoglossum officinale* L.			1
	Echium italicum subsp. biebersteinii (Lacaita) Greuter & Burdet			1
	*Heliotropium europaeum* L.			1
	*Lithospermum officinale* L.			
	*Myosotis ramosissima* Rochel			1
	*Myosotis sicula* Guss.			
** Brassicaceae **	*Alliaria petiolata* (M.Bieb.) Cavara & Grande			1
	*Alyssum chalcidicum* Janka	*	r	1
	*Arabidopsis thaliana* (L.) Heynh.			1
	*Arabis hirsuta* (L.) Scop.			
	*Aubrieta deltoidea* (L.) DC.			
	*Aurinia saxatilis* (L.) Desv.			
	*Calepina irregularis* (Asso) Thell.			1
	*Capsella bursa-pastoris* (L.) Medik.			1
	*Cardamine hirsuta* L.			1
	*Clypeola jonthlaspi* L.			1
	*Diplotaxis tenuifolia* (L.) DC.			1
	*Draba verna* L.			
	*Erysimum crassistylum* C. Presl			
	Hesperis laciniata All. subsp. laciniata			
	*Hirschfeldia incana* (L.) Lagr.-Foss.			1
	*Lepidium draba* L.			1
	Lunaria annua subsp. pachyrhiza (Borbás) Maire & Petitm.			1
	*Microthlaspi perfoliatum* (L.) F.K.Mey.			1
	*Rapistrum rugosum* (L.) All.			1
	*Sisymbrium officinale* (L.) Scop.			1
	*Teesdalia coronopifolia* (J.P.Bergeret) Thell.			
** Caesalpiniaceae **	*Cercis siliquastrum* L.			
** Campanulaceae **	*Asyneuma limonifolium* (L.) Janch.			
	*Campanula sparsa* Friv.	*		
	*Campanula versicolor* Andrews			
** Caprifoliaceae **	*Sambucus nigra* L.			1
** Caryophyllaceae **	*Cerastium glomeratum* Thuill.			1
	Holosteum umbellatum L. subsp. umbellatum			1
	*Petrorhagia dubia* (Raf.) G.López & Romo			1
	*Petrorhagia prolifera* (L.) P.W.Ball & Heywood			
	*Petrorhagia saxifraga* (L.) Link			
	*Silene graeca* Boiss. & Spruner	*	r?	
	*Silene latifolia* Poir.			1
	*Silene ungeri* Fenzl	*	r	
	*Silene vulgaris* (Moench) Garcke			1
	*Stellaria media* (L.) Vill.			1
** Celastraceae **	*Euonymus verrucosus* Scop.			
** Chenopodiaceae **	*Chenopodium album* L.			1
** Convolvulaceae **	*Calystegia sepium* (L.) R.Br.			
	*Convolvulus althaeoides* L.			1
	*Convolvulus arvensis* L.			1
	*Convolvulus cantabrica* L.			
	*Cuscuta campestris* Yunck.		X	1
** Crassulaceae **	*Sedum acre* L.			
	*Sedum album* L.			
	*Sedum annuum* L.			
	*Umbilicus horizontalis* (Guss.) DC.			
	*Umbilicus rupestris* (Salisb.) Dandy			
** Dipsacaceae **	*Pterocephalus plumosus* (L.) Coult.			
	*Scabiosa tenuis* Boiss.	*	r?	
** Euphorbiaceae **	*Euphorbia cyparissias* L.			1
	*Euphorbia helioscopia* L.			1
	*Euphorbia myrsinites* L.			
	Euphorbia platyphyllos L. subsp. platyphyllos			1
	*Mercurialis annua* L.			1
	*Mercurialis ovata* Sternb. & Hoppe			
** Fabaceae **	Anthyllis vulneraria subsp. bulgarica (Sagorski) Cullen	*		
	Colutea arborescens L. subsp. arborescens			
	*Galega officinalis* L.			1
	Hippocrepis emerus subsp. emeroides (Boiss. & Spruner) Lassen			
	*Lathyrus setifolius* L.			1
	*Lotus pedunculatus* Cav.			
	*Medicago arabica* (L.) Huds.			1
	*Medicago minima* (L.) Bartal.			1
	*Medicago polymorpha* L.			1
	Medicago sativa L. subsp. sativa			1
	Medicago sativa subsp. falcata (L.) Arcang.			1
	*Melilotus albus* Medik.			1
	*Melilotus officinalis* (L.) Lam.			1
	*Oxytropis pilosa* (L.) DC.			
	*Robinia pseudoacacia* L.		X	1
	*Spartium junceum* L.			1
	*Trifolium campestre* Schreb.			
	*Trifolium dubium* Sibth.			
	*Trifolium pratense* L.			
	*Trifolium repens* L.			1
	*Vicia sativa* L.			1
	Vicia villosa subsp. microphylla (d'Urv.) P.W.Ball			1
	Vicia villosa subsp. varia (Host) Corb.			1
** Fagaceae **	*Quercus frainetto* Ten.			
** Fumariaceae **	*Fumaria rostellata* Knaf			1
** Geraniaceae **	*Erodium ciconium* (L.) L'Hér.			1
	*Geranium lucidum* L.			1
	*Geranium molle* L.			1
	*Geranium purpureum* Vill.			1
	*Geranium rotundifolium* L.			1
** Hippocastanaceae **	*Aesculus hippocastanum* L.	*		
** Hyacinthaceae **	*Muscari comosum* (L.) Mill.			1
	*Muscari neglectum* Ten.			
	*Ornithogalum sibthorpii* Greuter			
	*Prospero autumnale* (L.) Speta			
** Hypericaceae **	*Hypericum perforatum* L.			1
	*Hypericum rumeliacum* Boiss.	*		
** Iridaceae **	Crocus cancellatus Herb. subsp. maziaricus (Herb.) B.Mathew			
	*Iris attica* Boiss. & Heldr.			
** Juglandaceae **	*Juglans regia* L.			
** Lamiaceae **	Ballota nigra subsp. sericea (Vandas) Patzak	*	r	1
	Lamium amplexicaule L.			1
	Lamium garganicum L. subsp. garganicum			
	*Marrubium peregrinum* L.			1
	*Mentha aquatica* L.			
	*Salvia candidissima* Vahl			
	Sideritis montana L. subsp. montana			1
	*Stachys annua* (L.) L.			1
	*Stachys plumosa* Griseb.	*		
	*Teucrium capitatum* L.			
	*Thymus longicaulis* C. Presl			
	*Thymus teucrioides* Boiss. & Spruner	*	r	
** Lauraceae **	*Laurus nobilis* L.			
** Liliaceae **	*Lilium candidum* L.			
** Lythraceae **	*Lythrum salicaria* L.			
** Malvaceae **	Alcea biennis subsp. cretica (Weinm.) Valdés	*		1
	*Malva sylvestris* L.			1
** Moraceae **	*Broussonetia papyrifera* (L.) Vent.		X	1
	*Ficus carica* L.			
	*Morus alba* L.		X	1
** Oleaceae **	*Jasminum fruticans* L.			
	*Phillyrea latifolia* L.			
** Orchidaceae **	*Ophrys helenae* Renz	*	r	
	*Ophrys sphegodes* Mill.			
** Orobanchaceae **	*Orobanche sp*			
** Papaveraceae **	*Chelidonium majus* L.			1
	*Papaver dubium* L.			1
	*Papaver rhoeas* L.			1
** Plantaginaceae **	*Plantago lanceolata* L.			1
	Plantago major L. subsp. major			1
** Platanaceae **	*Platanus orientalis* L.			
** Poaceae **	*Aegilops neglecta* Bertol.			1
	*Avena barbata* Link			1
	*Avena sterilis* L.			1
	*Bothriochloa ischaemum* (L.) Keng			
	*Bromus diandrus* Roth			1
	*Bromus sterilis* L.			1
	*Cynodon dactylon* (L.) Pers.			1
	*Dactylis glomerata* L.			1
	*Dasypyrum villosum* (L.) P.Candargy			1
	*Hordeum marinum* Huds.			
	Hordeum murinum subsp. leporinum (Link) Arcang.			1
	*Lolium perenne* L.			1
	*Lolium rigidum* Gaudin			1
	*Melica ciliata* L.			
	Melica transsilvanica subsp. klokovii Tzvelev			
	*Milium vernale* M. Bieb.			1
	*Phleum subulatum* (Savi) Asch. & Graebn.			
	*Piptatherum holciforme* (M. Bieb.) Roem. & Schult.			
	*Poa annua* L.			1
	*Poa bulbosa* L.			
	*Poa nemoralis* L.			
	*Poa trivialis* L.			
	*Setaria pumila* (Poir.) Roem. & Schult.			1
	*Setaria viridis* (L.) P. Beauv. subsp. viridis			1
	*Stipa thessala* Hausskn.	*	r	
** Polygonaceae **	Rumex acetosella subsp. acetoselloides (Balansa) Nijs			1
	*Rumex conglomeratus* Murray			
	*Rumex crispus* L.			1
** Portulacaceae **	*Portulaca oleracea* L. s.l.			1
** Primulaceae **	*Cyclamen hederifolium* Aiton			
	*Lysimachia vulgaris* L.			
** Ranunculaceae **	*Anemone pavonina* Lam.			
	*Clematis flammula* L.			
	*Delphinium balcanicum* Pawł.	*	r	1
	*Delphinium peregrinum* L.			1
	*Nigella damascena* L.			1
	*Ranunculus psilostachys* Griseb.	*		
** Rhamnaceae **	*Paliurus spina-christi* Mill.			
** Rosaceae **	*Crataegus monogyna* Jacq.			
	*Prunus avium* (L.) L.			
	Prunus domestica subsp. insititia (L.) Bonnier & Layens			1
	*Prunus dulcis* (Mill.) D.A. Webb		X	1
	Prunus spinosa subsp. dasyphylla (Schur) Domin			
	*Prunus webbii* (Spach) Vierh.			
	*Pyracantha coccinea* M. Roem.			
	*Rosa canina* L.			
	*Rubus canescens* DC.			
	*Rubus sanctus* Schreb.			1
	*Sanguisorba minor* Scop.			
** Rubiaceae **	*Galium aparine* L.			1
	Galium setaceum subsp. decaisnei (Boiss.) Ehrend.			
	*Galium spurium* L.			1
	Galium verum L. subsp. verum			
** Ruscaceae **	*Ruscus aculeatus* L.			
** Rutaceae **	*Dictamnus albus* L.			
** Salicaceae **	*Populus alba* L.			
	*Populus nigra* L.			
** Saxifragaceae **	*Saxifraga tridactylites* L.			1
** Scrophulariaceae **	*Linaria dalmatica* (L.) Mill.			1
	Linaria genistifolia (L.) Mill. subsp. genistifolia			1
	*Linaria peloponnesiaca* Boiss. & Heldr.	*		1
	*Scrophularia heterophylla* Willd.			
	*Verbascum graecum* Boiss.	*	r?	1
	*Verbascum undulatum* Lam.	*		1
** Simaroubaceae **	*Ailanthus altissima* (Mill.) Swingle		X	1
** Smilacaceae **	*Smilax excelsa* L.			
** Tiliaceae **	*Tilia rubra* DC.			
** Ulmaceae **	*Celtis australis* L.			
	*Ulmus minor* Mill.			
** Urticaceae **	*Parietaria cretica* L.			
	*Parietaria officinalis* L.			
	*Urtica dioica* L.			1
** Valerianaceae **	*Valeriana italica* Lam.			
	*Valerianella echinata* (L.) DC.			1
	*Valerianella locusta* (L.) Laterr.			1
** Veronicaceae **	*Veronica hederifolia* L.			1
	*Veronica polita* Fr.			1
	*Veronica triloba* (Opiz) Opiz			1
**Vervenaceae**	*Verbena officinalis* L.			1
